# The effectiveness of contracting-out of human resources for health in health service

**DOI:** 10.1186/1471-2458-14-S1-O3

**Published:** 2014-01-29

**Authors:** Dwi Handono Sulistyo, Laksono Trisnantoro, Tjahjono Koentjoro

**Affiliations:** 1 Faculty of Medicine, Universitas Gadjah Mada, Yogyakarta 55281, Indonesia

## Background

Although contracting out initiatives had been carried out in various countries, this policy has not been a major issue in Indonesia. As a result, efforts to address health problems, especially in remote areas, continue to use the conventional approach which is through the appointment of civil servants. In this situation the three regions of Indonesia took the initiative to do some contracting out of health services and to overcome the shortage of health problems. The objective of this study was to assess the effectiveness of such contracting out initiatives.

## Materials and methods

Case study was conducted in Kelay Health Center, Berau (East Kalimantan), Gunungsitoli Local Hospital, Nias (North Sumatra), and Six district hospitals in the Province of East Nusa Tenggara. Data were collected through interviews, observations and secondary documents. Data was analysed by means of descriptive and statistical methods. Analysis tool used was programme-level logic model and cross-case synthesis.

## Results

Initiatives in Berau failed to proceed due to the absence of private providers that met the criteria, while the initiative in Nias and NTT was successfully implemented. Both in Nias and in NTT, the initiative managed to overcome a shortage of certain health workers, and improved access and quality of care. Even in NTT, the number of maternal and neonatal deaths and the number of intrauterine foetal deaths were absolutely reduced.

## Conclusions

Access and quality of care was successfully improved through contracting out initiatives in Berau and NTT. Also, both initiatives were successful in overcoming a shortage of certain human resources for health in both areas.

